# Breaking the Trade‐Off of Mechanical Robustness and Energy Storage Capacity in Phase Change Materials Through a Molecular Design Strategy of Hard‐Segment‐Anchored/Side‐Chain‐Storage

**DOI:** 10.1002/advs.76170

**Published:** 2026-06-15

**Authors:** Huizhou Luo, Hebo Shi, Jun Zhang, Henghui Deng, Yongyin Zhu, Zehong Chen, Chaoqun Zhang

**Affiliations:** ^1^ College of Future Biomass South China Agricultural University Guangzhou China; ^2^ College of Material Science and Art Design Inner Mongolia Agricultural University Hohhot China

**Keywords:** flexible, phase change materials, polyurethane, thermal management

## Abstract

Phase change materials (PCMs) have garnered significant attention for personal thermal management, yet conventional solid‐liquid PCMs suffer from potential leakage issues, and existing solid‐solid polyurethane‐based PCMs (PU‐PCMs) often face an inherent trade‐off between mechanical robustness and energy storage capacity. Here, we develop a molecular design strategy of hard‐segment‐anchored/side‐chain‐storage to decouple these competing functions by relocating the phase‐change units from the polymer main chain to side chains. Specifically, a series of flexible side‐chain PU‐PCMs were synthesized using 2,2'‐(octadecylimino)diethanol (SDEA) bearing crystallizable C18 alkyl side chains and 1,4‐butanediol (BDO) as chain extender. The hard segments formed by BDO and hexamethylene diisocyanate provide a robust physical network, while the hanging C18 side chains independently crystallize to enable solid‐solid phase transition and latent heat storage. By adjusting the BDO‐to‐SDEA molar ratio, the optimized material, SPU‐BDO_0.20_, exhibits a desirable combination of high toughness, excellent flexibility (elongation at break >600%), high phase change enthalpy (51.1 J g^−^^1^), and outstanding thermal cycling stability (500 cycles). Notably, electrospun fibrous membranes fabricated from SPU‐BDO_0.20_ demonstrate effective temperature regulation, maintaining a surface temperature 3°C–4°C lower than cotton controls under heating. This work provides a new strategy for developing high‐performance, flexible, and leakage‐free PCMs for wearable thermal management applications.

## Graphical abstract

To decouple mechanical robustness and energy storage capacity in phase change materials (PCMs), a series of polyurethane‐based PCMs bearing crystallizable alkyl side chains are synthesized via a hard‐segment‐anchored/side‐chain‐storage molecular design. The optimized material achieves a desirable combination of elongation at break of 647.5%, toughness of 26.9 MJ m^−^^3^, and phase‐change enthalpy of 51.1 J g^−^^1^ with outstanding stability over 500 cycles.

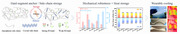

## 1 Introduction

Phase change materials (PCMs) have garnered significant attention for their ability to store and release latent heat during reversible phase transitions, offering transformative potential in energy management systems [1, 2, 3]. Among them, solid‐solid (S‐S) PCMs are particularly attractive due to their leakage‐free operation and structural stability during cycling, making them promising candidates for advanced thermal regulation applications [4, 5, 6]. To address the inherent limitations of traditional PCMs, such as low thermal cycling stability and poor mechanical robustness, three primary strategies have been developed: polymer blending [7, 8], microencapsulation [9, 10], and intrinsic cross‐linking [11, 12, 13]. While blending enhances processability and microencapsulation prevents leakage, intrinsic cross‐linking emerges as a superior approach by integrating phase change units directly into a covalently bonded polymer network. This strategy simultaneous achieves shape stability, mechanical elasticity, and scalable processability.

Polyurethane‐based PCMs (PU‐PCMs) represent a prominent class of intrinsically flexible S‐S PCMs [14, 15]. To date, most reported PU‐PCMs have utilized polyethylene glycol (PEG) as the soft segment, where the crystallizable PEG chains also serve as the phase change component. For example, by chemically cross‐linking 4‐aminophenyl disulfide and hexamethylene diisocyanate with PEG, the obtained polyurethane film had tunable phase change temperatures (from 38.8°C to 51.1°C) and enthalpy (from 79.7 to 116.7 J g^−1^), demonstrating applications in synchronous visual/infrared stealth [16]. Similarly, PEG/4,4′‐methylenebis(cyclohexyl isocyanate) solid‐solid phase change fibers were prepared by polycondensation and a wet‐spinning process, demonstrating promising potential for wearable intelligent thermal management [17]. Furthermore, flexible PCM films were prepared via a general polymerization strategy of synthesizing intrinsic PCM films by polymerically chemical grafting of melamine and toluene‐2,4‐diisocyanate with PEG. The PCM films exhibited tunable phase transition temperatures with different PEG molecular weights, relatively high latent heat, and mechanical flexibility, holding great potential for next‐generation flexible thermal management electronics [18]. Despite these advances, the above systems rely on PEG as the main‐chain phase change component, which imposes an inherent trade‐off: higher PEG content enhances enthalpy but compromises mechanical strength, while lower PEG content improves strength at the expense of energy storage capacity.

**FIGURE 1:**
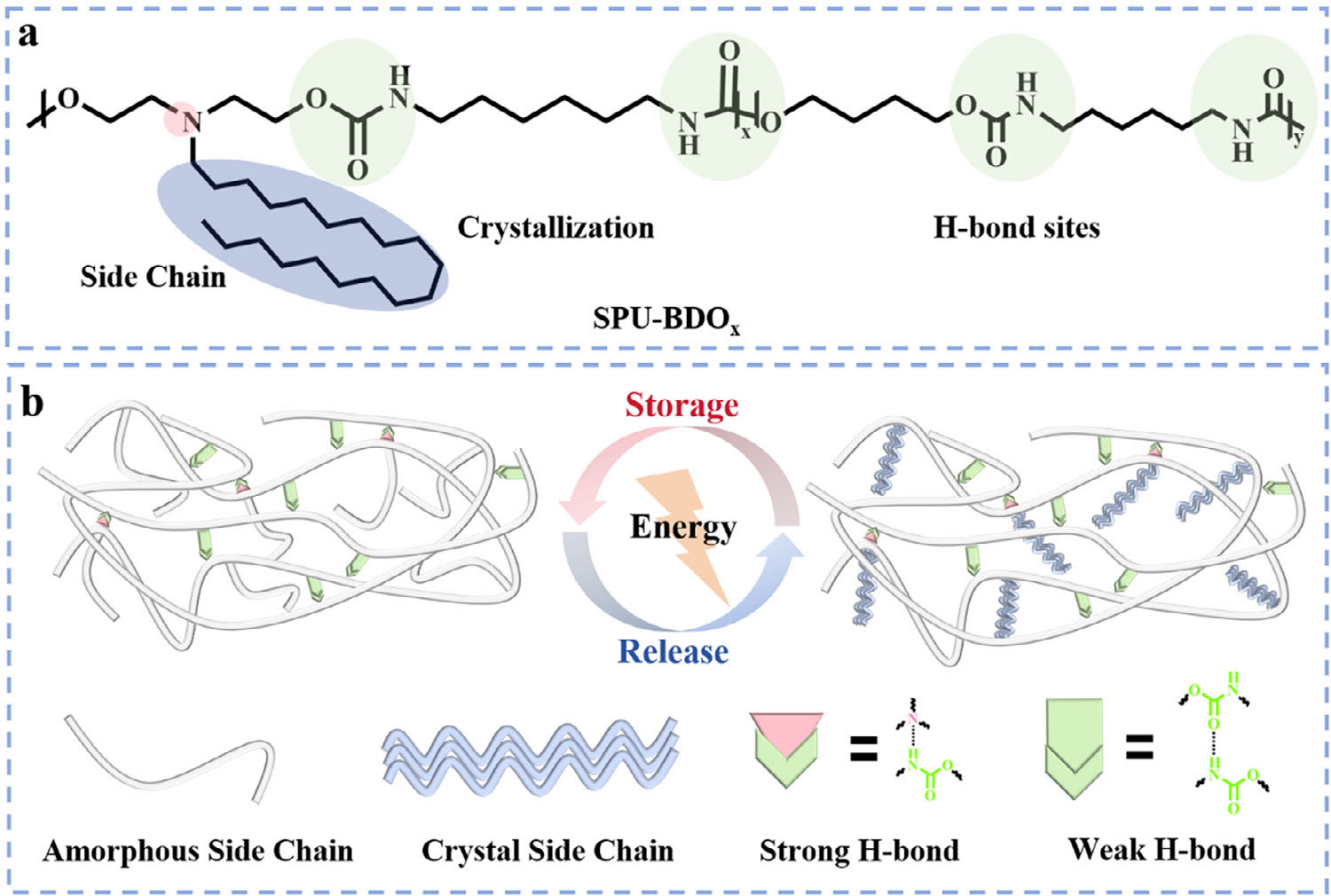
(a) The chemical structure of SPU‐BDO_x_. (b) Schematic illustration of the working mechanism of SPU‐BDO_x_ on thermal energy storage and release.

Herein, we propose a molecular design strategy to decouple and synergistically optimize mechanical properties and energy storage capacity. As shown in Figure 1a, we introduce a “hard‐segment‐anchored/side‐chain‐storage” architecture, where the phase change energy storage units are relocated from the main chain to side chains, thereby separating the mechanical‐supporting framework from the phase‐change domains. Specifically, a series of side‐chain polyurethane‐based PCMs (SPU‐BDO_x_) were synthesized via stepwise polymerization using 2,2'‐(octadecylimino)diethanol (SDEA) bearing crystallizable C18 alkyl side chains as the phase change component, 1,4‐butanediol (BDO) as the chain extender, and hexamethylene diisocyanate (HDI) as the hard‐segment building block. In this design, the hard segments formed by BDO and HDI provide a robust physical network that mechanically supports the material and prevents macroscopic flow even above the melting temperature, while the hanging C18 side chains independently crystallize to enable solid‐solid phase transition and latent heat storage (Figure 1b). Such a side‐chain‐storage architecture allows the C18 chains to crystallize and melt without substantially disrupting the backbone integrity, thus reducing the mechanical deterioration commonly associated with main‐chain crystallization. By adjusting the BDO‐to‐SDEA molar ratio, the density of hard segments and hydrogen bonding can be precisely tuned, thereby balancing mechanical performance with phase change capacity. The optimized material, SPU‐BDO_0.20_ film, exhibits a desirable combination of high toughness, excellent flexibility (elongation at break >600%), stable phase change enthalpy (51.1 J g^−^^1^) near body temperature, and outstanding thermal cycling stability. Finally, the practical potential of SPU‐BDO_0.20_ for wearable thermal management is demonstrated via an electrospun fibrous membrane, which achieves effective temperature regulation under both simulated solar irradiation and on‐skin conditions. This work provides a new molecular design paradigm for developing high‐performance, flexible, and leakage‐free PCMs for passive thermal‐buffering coating and wearable thermal management.

## 2 Results and Discussion

### 2.1 Preparation and Characterization of SPU‐BDO_x_

**FIGURE 2:**
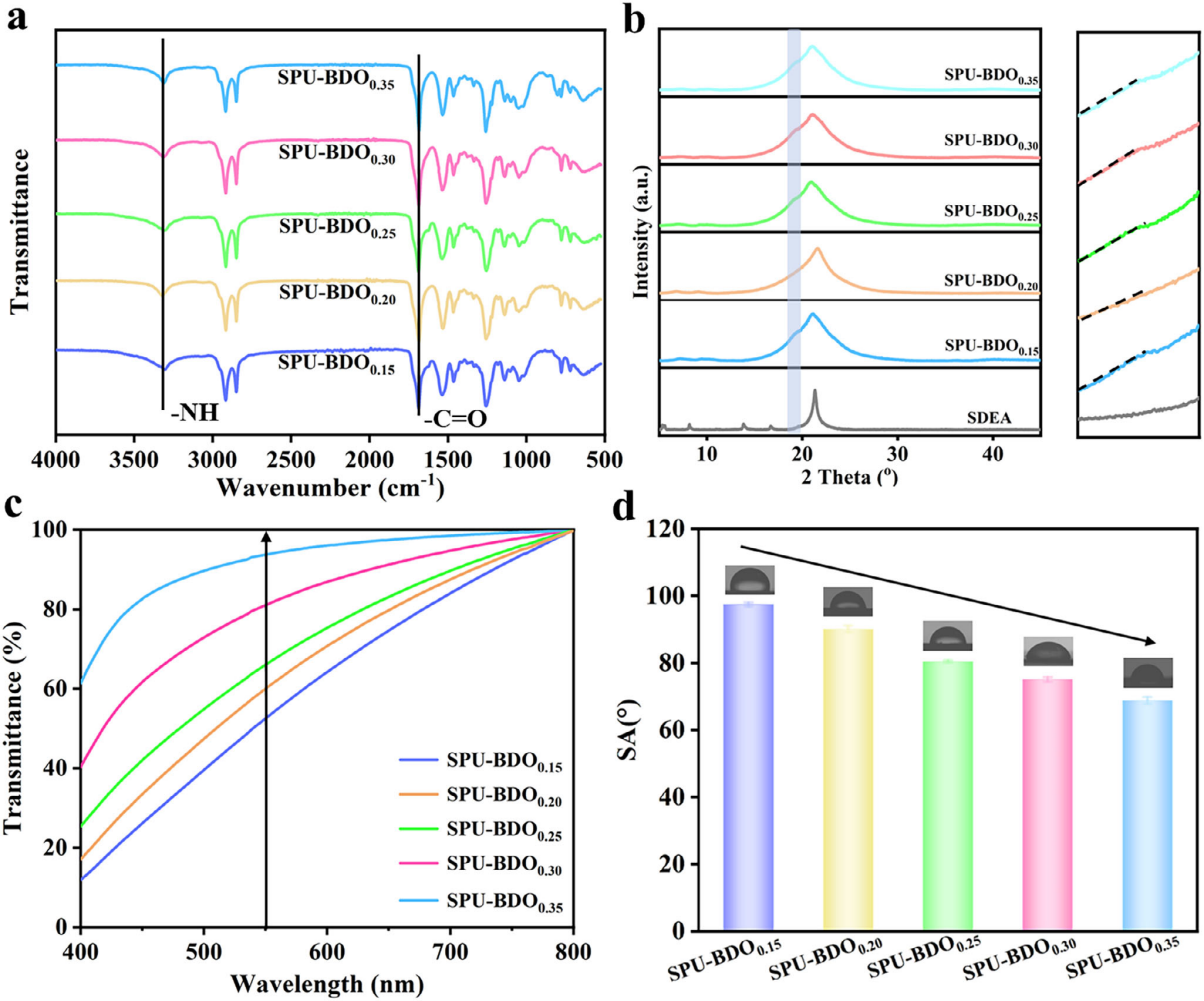
(a) ATR‐FTIR spectra of SPU‐BDO_x_. (b) XRD patterns of SPU‐BDOx and partial enlargement of 18°–20°. (c) Transmittance spectra of SPU‐BDO_x_. (d) Water contact angles of SPU‐BDO_x_. Inset: photographs of contact angles.

The chemical structure of the prepared SPU‐BDO_x_ was characterized using ATR‐FTIR, ^1^H NMR, and GPC. As shown in Figure 2a, no characteristic peaks of ‐NCO can be detected near 2265 cm^−1^ band for all samples, indicating the complete consumption of ‐NCO groups during polymerization. Meanwhile, the characteristic vibrational modes of typical polyurethane structures are clearly observed. The broad absorption at 3335 cm^−1^ is attributed to the stretching vibration of the ─N─H bond of urethane, and the sharp peak at 1696 cm^−1^ corresponds to the stretching vibration of ─C═O. The ^1^H NMR spectrum of SPU‐BDO_0.20_ further confirms the formation of a polyurethane structure (Figure S1). The molecular weight of SPU‐BDO_x_ was characterized using GPC. As shown in Figure S2 and Table S1, all SPU‐BDO_x_ exhibits similar peak positions, suggesting comparable molecular weights.

The crystallization behavior, which is crucial for phase change energy storage, was investigated by XRD. As shown in Figure 2b, SDEA monomer shows a sharp diffraction peak at 2*θ* ≈ 21.3°, consistent with the (110) plane of octadecane (PDF# 00‐001‐0786), confirming the ordered packing of its C18 alkyl chain. In contrast, the SPU‐BDO_x_ polymers show a much broader diffraction peak centered around 2θ ≈ 21.1°. This indicates that while the C18 side chains retain their ability to crystallize, their packing is significantly disrupted and confined within the thermoplastic polyurethane network. The broadened crystallization peak confirms that the phase change capability originates from the C18 side‐chain domains rather than the hard segments. This side‐chain crystallinity is the fundamental basis for the material's latent heat storage. The weak shoulder peak at 2*θ* ≈ 19.3° is mainly assigned to less‐ordered or imperfect crystalline domains of the C18 side chains confined within the polyurethane matrix. Although local ordering of hydrogen‐bonded hard segments may contribute to the broad scattering background, the absence of independent sharp diffraction peaks from the BDO/HDI hard segments suggests that this shoulder primarily originates from imperfect alkyl side‐chain packing rather than distinct hard‐segment crystallization.

The crystallinity and composition of the SPU‐BDO_x_ films directly influence their macroscopic optical and surface properties. The transparency of SPU‐BDO_x_ films was evaluated via UV transmittance. As shown in Figure 2c, the transmittance of SPU‐BDO_x_ increases significantly with the increase of BDO content, ascribed to reduced SDEA fraction and consequently diminished crystallinity. Meanwhile, the surface hydrophobicity was assessed by water contact angle measurement (Figure 2d). All films have water contact angles exceeding 60°, with values of 97.5°, 90.2°, 80.4°, 75.1°, and 68.8° for SPU‐BDO_0.15_, SPU‐BDO_0.20_, SPU‐BDO_0.25_, SPU‐BDO_0.30_ and SPU‐BDO_0.35_, respectively. The decreasing water contact angle is attributed to the reduced SDEA content, whose long hanging side chain C18 structure can provide excellent hydrophobicity.

### 2.2 Mechanical Properties and Toughening Mechanism of SPU‐BDO_x_

**FIGURE 3:**
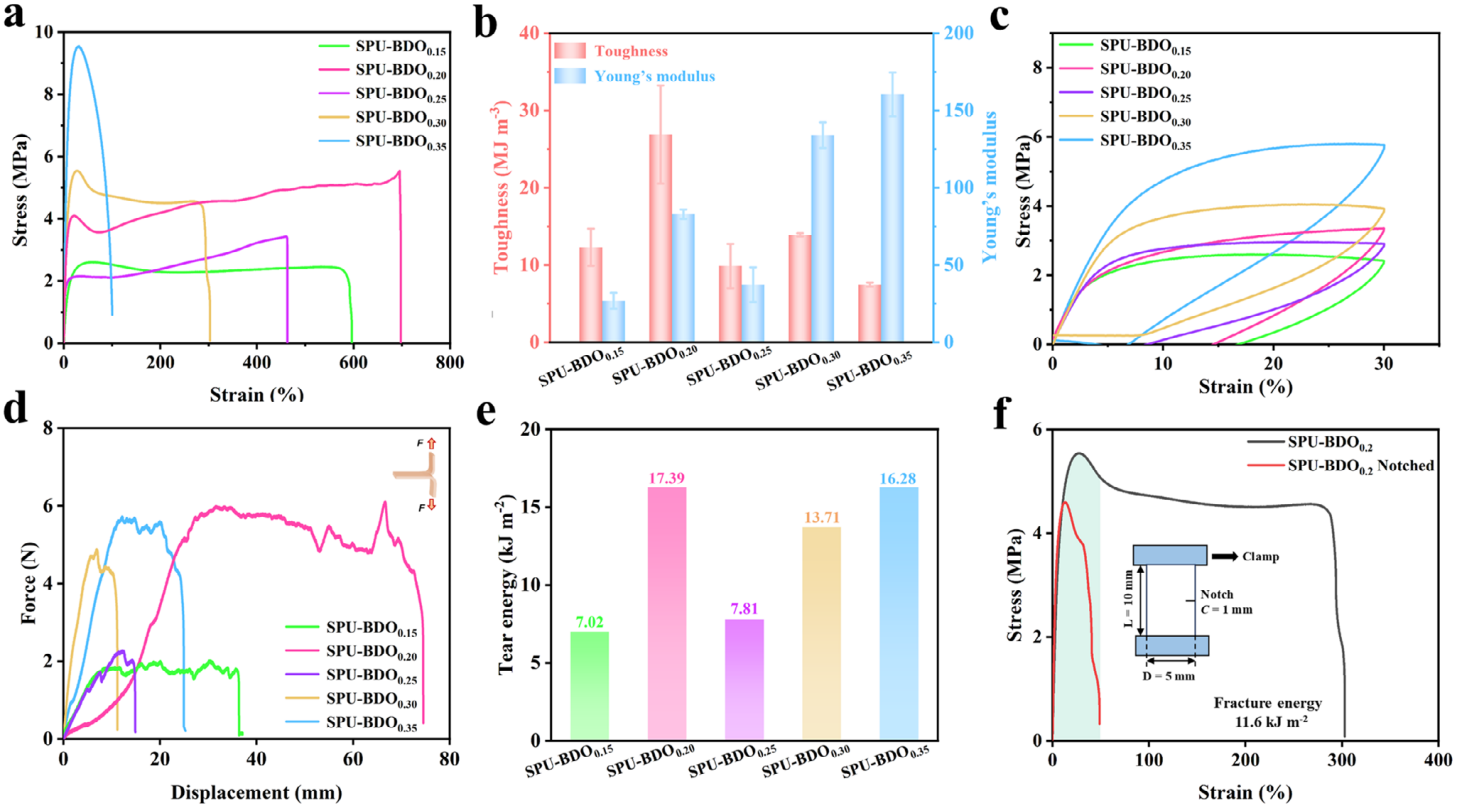
(a) Stress‐strain curves and (b) toughness and Young's modulus. (c) Cyclic loading‐unloading tensile curves with a maximum strain of 30%. (d) Force‐displacement curves measured by the tearing test and (e) the corresponding tearing energy. (f) Stress‐strain curves of unnotched and notched samples, measured at the strain rate of 20 mm min^−1^.

To investigate the effect of BDO‐to‐SDEA molar ratio on the mechanical properties, all polyurethanes were subjected to tensile testing using a universal tensile machine. Figure 3a shows the stress‐strain curves, and the corresponding data including tensile strength, elongation at break, Young's modulus, and toughness are summarized in Table S2. As the BDO molar ratio increases from 0.15 to 0.35, the tensile strength of SPU‐BDO_x_ increases from 2.5 to 10.3 MPa, while the Young's modulus increases from 26.8 to 160.4 MPa (Figure 3b). The enhancement in tensile strength and Young's modulus is mainly attributed to the increased formation of urethane‐based hard segments from BDO and HDI, which improves structural rigidity and hydrogen‐bonding density. In contrast, the elongation at break and toughness first increase and then decrease with higher BDO content. At relatively low BDO content, the abundant flexible C18 side chains favor chain mobility and deformation, whereas the moderate introduction of BDO enhances hard‐segment density and energy dissipation. Therefore, SPU‐BDO_0.20_ achieves the best overall mechanical performance, with a tensile strength of 5.9 MPa, an exceptional elongation at break of 647.5%, and a toughness of 26.9 MJ m^−3^. It is worth noting that SPU‐BDO_0.25_ shows a local deviation from the overall composition‐dependent trend, with lower elongation at break and toughness than SPU‐BDO_0.15_ and SPU‐BDO_0.20_. This non‐monotonic behavior may be associated with a less efficient balance between chain mobility and hard‐segment reinforcement. Specifically, the reduced SDEA content weakens the flexible C18 side‐chain contribution, while the BDO/HDI‐derived hard segments may not yet be sufficiently organized to provide effective reinforcement, thus leading to less efficient stress transfer and reduced energy dissipation. However, when the BDO content is further increased to 0.30 and 0.35, the higher hard‐segment density results in higher modulus and strength, but also restricts chain extension and thus reduces ductility and toughness. Consequently, an optimal BDO content is essential to balance these properties. Furthermore, the increasing BDO content leads to a rise in the hysteresis of SPU‐BDO_x_ (Figure 3c), indicating elevated energy dissipation during cyclic loading.

Tear resistance, an important mechanical property, reflects a material's capacity to withstand large external forces without rapid failure. The tearing test reveals that SPU‐BDO_0.20_ achieves the highest tear energy of 17.39 kJ m^−2^ (Figure 3d,e). To evaluate the fracture energy, the optimum sample SPU‐BDO_0.20_ was subjected to a uniaxial notched tensile test. The 1 mm notched SPU‐BDO_0.20_ can be stretched up to 50% strain, confirming its excellent mechanical reliability even under severely damaged conditions (Figure 3f).

**FIGURE 4:**
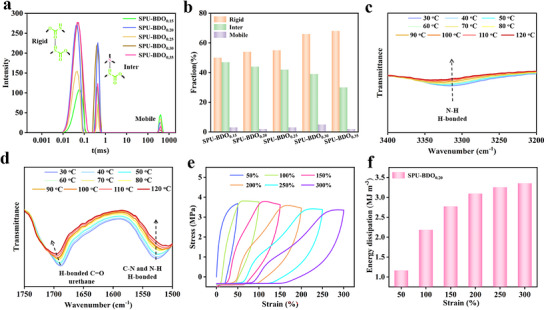
(a) Inverted low‐field NMR spectra of SPU‐BDO_x_ and (b) the corresponding distribution of rigid, intermediate, and mobile components. Variable‐temperature FTIR of SPU‐BDO_0.20_ at (c) 3400–3250 cm^−1^ and (d) 1750–1500 cm^−1^. (e) Sequential loading‐unloading tensile curves of SPU‐BDO_0.20_ and (f) the corresponding dissipated energy in each cycle.

The mechanical properties, particularly toughness, of the SPU‐BDO_x_ films are predominantly governed by their hydrogen‐bonding network. In polyurethanes, urethane groups within the hard segments act as physical cross‐linking points via hydrogen bonds, which restrict chain mobility and leads to shorter transverse relaxation times (*T*_2_). Conversely, lone pair electrons from tertiary amine may form weak hydrogen bonds with adjacent carbamates, leading to an increase in *T*_2_ value for the medium fraction. Therefore, by measuring the *T*_2_ values of different components via low‐field NMR analysis, it is possible to distinguish different phase regions and assess the strength of hydrogen bonds. The transverse relaxation decay curves were analyzed by fitting a linear combination of Gaussian, Weibull, and exponential functions, as described by the following Equation (1):

(1) $$\begin{array}{cc} f\;\left(t\right)= & f_{r}\;\;exp(-\left(t/T_{2rigid^{2}}\right)+f_{i}\;exp(-{\left(t/T_{2inter}\right)}^{v_{i}}\\ & +f_{m}\;exp\left(-\left(t/T_{2mobile}\right)\right) \end{array}$$

where *f_r_*, *f_i_*, and *f_m_* are the proportions of rigid, intermediate, and mobile components, corresponding to the apparent relaxation times *T*_2rigid_, *T*_2inter_, and *T*_2mobile_, respectively. These components reflect restricted motion with high hydrogen bond density, intermediate mobility with partial hydrogen bond participation, and high mobility without significant hydrogen bond limitation, respectively [19]. As shown in Figure 4a, the relaxation time distributions of SPU‐BDO_x_ remain consistent, ranging from 0.05–350 ms. Figure 4b summarizes the percentage of different hydrogen bonds in SPU‐BDO_x_. It can be found that increasing the BDO content from 0.15 to 0.35 significantly raises the proportion of the rigid component (*f_r_*). SPU‐BDO_0.35_ with the highest BDO content has the highest *f_r_* component. This directly aligns with their mechanical properties, with SPU‐BDO_0.35_ showing the highest Young's modulus and fracture strength [20]. Overall, the increase of BDO content leads to the formation of more urethane‐based hard segments, which provide more hydrogen bonding sites, finally resulting in the elevation of hydrogen bonding density [21]. Such controllable chemical structure and hydrogen bonding density can directly influence the mechanical properties. Specifically, both fracture elongation and toughness initially increase with BDO content. However, beyond an optimal BDO content, excessive hard‐segment density restricts chain mobility, causing reduced toughness and elongation at break [22].

To further investigate the dynamic nature of these hydrogen bonds, variable‐temperature FTIR spectroscopy was performed on SPU‐BDO_0.20_. As shown in Figure 4c, upon heating from 30 to 120°C, the stretching vibration of the ‐N‐H group (3200‐3500 cm^−1^) shifts to a higher wavenumber with diminished intensity. The ─C═O stretching vibration (1750–1500 cm^−1^) also undergoes a slight redshift (Figure 4d). This is mainly due to the intensification of molecular motion caused by the increased temperature, which leads to changes in the intermolecular hydrogen bonding interactions. These changes reflect the dissociation and reconstruction of hydrogen bonding dynamics in polyurethane molecules [23].

The reversible breakage and reorganization of hydrogen bonds has a significant impact on energy dissipation capability [24]. A cyclic tensile test was performed on SPU‐BDO_0.20_ with sequential strain from 50% to 300%. As shown in Figure 4e, the hysteresis loop area increases with the cyclic loading process. The corresponding dissipated energy calculated by integrating these loops increases significantly from 1.16 MJ m^−3^ at 50% strain to 3.25 MJ m^−3^ at 250% strain, and then remains nearly constant. (Figure 4f). To further quantify the energy‐dissipation capability, the damping efficiency was calculated from each loading‐unloading cycle. As shown in Figure S4, SPU‐BDO_0.20_ exhibits damping efficiencies of 82.3%, 72.7%, 76.4%, 75.8%, 77.3%, and 76.6% at strains of 50%, 100%, 150%, 200%, 250%, and 300%, respectively. These results demonstrate that the reversible hydrogen‐bonded physical associations effectively dissipate mechanical energy during deformation. This was due to the fact that, at lower strains, hydrogen bonds rupture and dissipate energy; as cycling continues, the system reaches a stable structure or relative equilibrium state where bond reformation rates match dissociation, leading to stable energy dissipation.

Overall, the toughening mechanism of SPU‐BDO_x_ can be attributed to a well‐tailored network, modulated by the BDO content. As evidenced by low‐field NMR, increasing BDO raises the proportion of rigid, hydrogen‐bond‐rich domains, enhancing modulus and strength. Meanwhile, variable‐temperature FTIR confirms the dynamic and reversible dissociation and reconstruction nature of these hydrogen bonds, which is crucial for energy dissipation. An optimal BDO content, as in BDO_0.20_, strikes a critical balance—providing sufficient hydrogen‐bonding sites for strength while maintaining adequate chain mobility for extensibility, resulting in the combination of excellent elongation, high toughness, and good elastic recovery [25].

### 2.3 Solid‐Solid Phase Transition Behaviors and Thermal Stability of SPU‐BDO_x_

**FIGURE 5:**
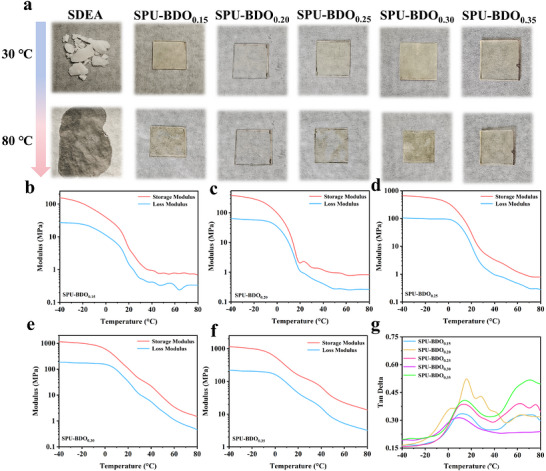
(a) Photographs of SPU‐BDO_x_ leakage test at 30°C and 80°C. Storage and loss modulus curves of (b) SPU‐BDO_0.15_, (c) SPU‐BDO_0.20_, (d) SPU‐BDO_0.25_, (e) SPU‐BDO_0.30_, and (f) SPU‐BDO_0.35_. (g) Tan delta curves of SPU‐BDO_x._

The shape‐stability and leakage resistance of the phase change materials were first evaluated. As shown in Figure 5a, upon heating from 30°C to 80°C, pure SDEA monomer melts completely, loses its shape, and penetrates the filter paper. In contrast, all SPU‐BDO_x_ films maintain their original shape without any leakage, and only have a slight change in transparency, from partially opaque to completely transparent. This visual change is attributed to the melting of the crystalline C18 side chains, since the grains in semi‐crystalline polymers may hinder the passage of light, whereas amorphous polymers provide non‐destructive light transmission [26]. This result demonstrates that the polymerization network of polyurethane can effectively constrain the flow of molten SDEA, ensuring leakage‐free, solid‐solid phase transition behavior. In addition, the phase transition behavior was further analyzed by DMA. As shown in Figure 5b–g, for all SPU‐BDO_x_, the energy storage modulus (E′) remains greater than the loss modulus (E″) throughout the heating process from −40°C to 80°C. This indicates that the synthesized SPU‐BDO_x_ had excellent solid‐state properties at temperatures lower than 80°C, which is also consistent with the leakage experiment results. Meanwhile, a sharp decrease in both E′ and E″ is observed around the temperature of 20°C, attributing to the phase transition of the C18 side chain from crystalline to amorphous state.

**FIGURE 6:**
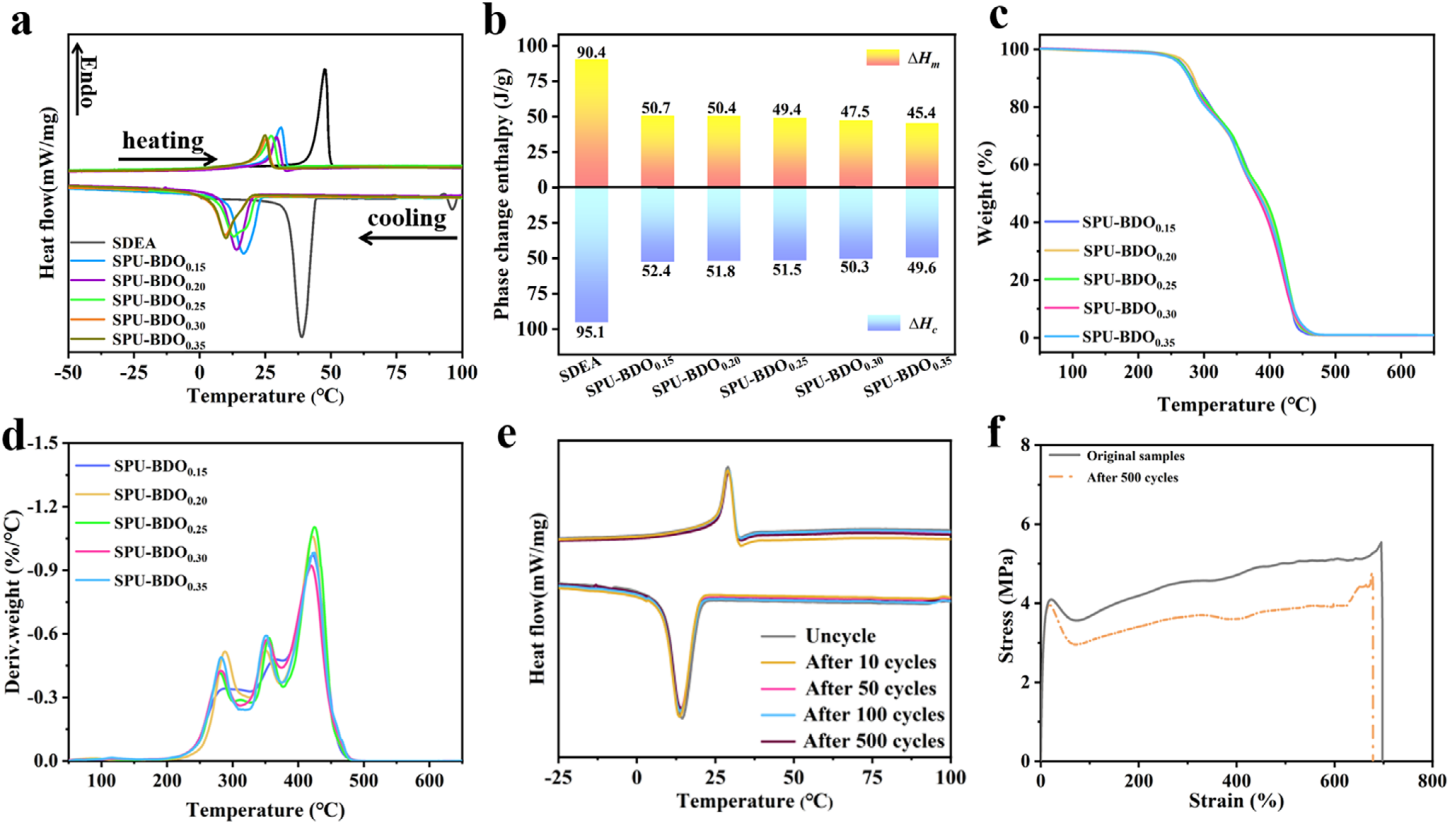
(a) DSC curves and (b) the corresponding phase transition enthalpies of SDEA and SPU‐BDO_x_. (c) TG and (d) DTG curves of SPU‐BDO_x_. (e) DSC curves of SPU‐BDO_0.20_ after different heating‐cooling cycles and (f) tensile stress‐strain curves of SPU‐BDO_0.20_ before and after 500 cycles.

The phase transition temperature and enthalpy of pure SDEA and SPU‐BDO_x_ were quantified by DSC, as shown in Figure 6a and the corresponding data were presented in Table S3. The upper half in the curves shows the heat absorption during the phase change heating process, while the lower half shows the exothermic curves during the phase change cooling process. All DSC curves have a heat absorption peak and an exothermic peak. SDEA shows a melting point of 46.7°C, a crystallization point of 38.4°C, and a high phase transition enthalpy of 92.8 J g^−1^. SPU‐BDO_x_ show similar phase transition profiles to that of SDEA, but both the melting and crystallization temperatures are reduced. This is attributed to restricted C18 side chain arrangement and decreased crystallinity, resulting from the backbone rigidity and restricted network of the polyurethane backbone [27]. In addition, the phase transition enthalpies decrease slightly with increasing BDO content, stabilizing around 50.0 J g^−1^ (Figure 6b). Among various samples, SPU‐BDO_0.20_ has a phase transition enthalpy of 51.1 J g^−1^. As summarized in Figure S3 and Table S5, many reported flexible PCMs exhibit either high latent heat with limited mechanical robustness or excellent elasticity with compromised energy‐storage density. By contrast, SPU‐BDO_0.20_ simultaneously delivers high elongation at break of 647.5% and a phase‐change enthalpy of 51.1 J g^−1^, demonstrating a balanced combination of mechanical flexibility and latent‐heat storage capacity enabled by the side‐chain‐storage strategy. Notably, the phase transition temperature range of 25°C–31°C is ideal for human thermal comfort, highlighting its potential application as a human comfort temperature‐controlled textile [28]. It should be noted that SPU‐BDO_0.20_ exhibits a certain degree of crystallization supercooling, with a melting temperature of approximately 29.2°C and a crystallization temperature of approximately 14.1°C. Therefore, SPU‐BDO_0.20_ is more suitable for intermittent personal thermal management scenarios, where heat absorption occurs near the human comfort temperature and thermal recharging can take place under cooler ambient conditions. Future molecular optimization, such as introducing nucleating motifs or regulating side‐chain packing, may further reduce supercooling and improve continuous cycling efficiency.

The thermal decomposition stability and long‐term cycling stability are critical for practical applications. The TG curves and derivative thermogravimetry (DTG) curves show that all SPU‐BDO_x_ samples have excellent thermal stability up to approximately 200°C, with the major decomposition step occurring above 250°C (Figure 6c,d). This confirms that SPU‐BDO_x_ possess sufficient thermal stability for applications within their intended operating temperature range. To evaluate the long‐term thermal cycling stability, SPU‐BDO_0.20_ was selected as a representative sample for further investigation based on its balanced mechanical and thermal properties. 500 heating‐cooling cycles between −25°C and 100°C were performed on SPU‐BDO_0.20_. As shown in Figure 6e, the melting and crystallizations curves show negligible change at first, 10th, 50th, 100th, and 500th cycles. In addition, the calculated melting and solidification enthalpies also remain essentially unchanged (Figure 6f and Table S4), indicating excellent cyclic stability of the phase change properties. After 500 heating‐cooling cycles, SPU‐BDO_0.20_ still retains a melting enthalpy of approximately 48.8 J g^−1^ and a crystallization enthalpy of approximately 50.6 J g^−1^, corresponding to retention ratios of 96.8% and 97.7%, respectively. Meanwhile, the tensile strength, elongation at break, toughness, and Young's modulus remain at approximately 4.8 MPa, 679.6%, 24.2 MJ m^−3^, and 78.6 MPa, respectively, confirming the excellent thermal‐mechanical durability of SPU‐BDO_0.20_.

### 2.4 Thermal Regulation and Management Ability

**FIGURE 7:**
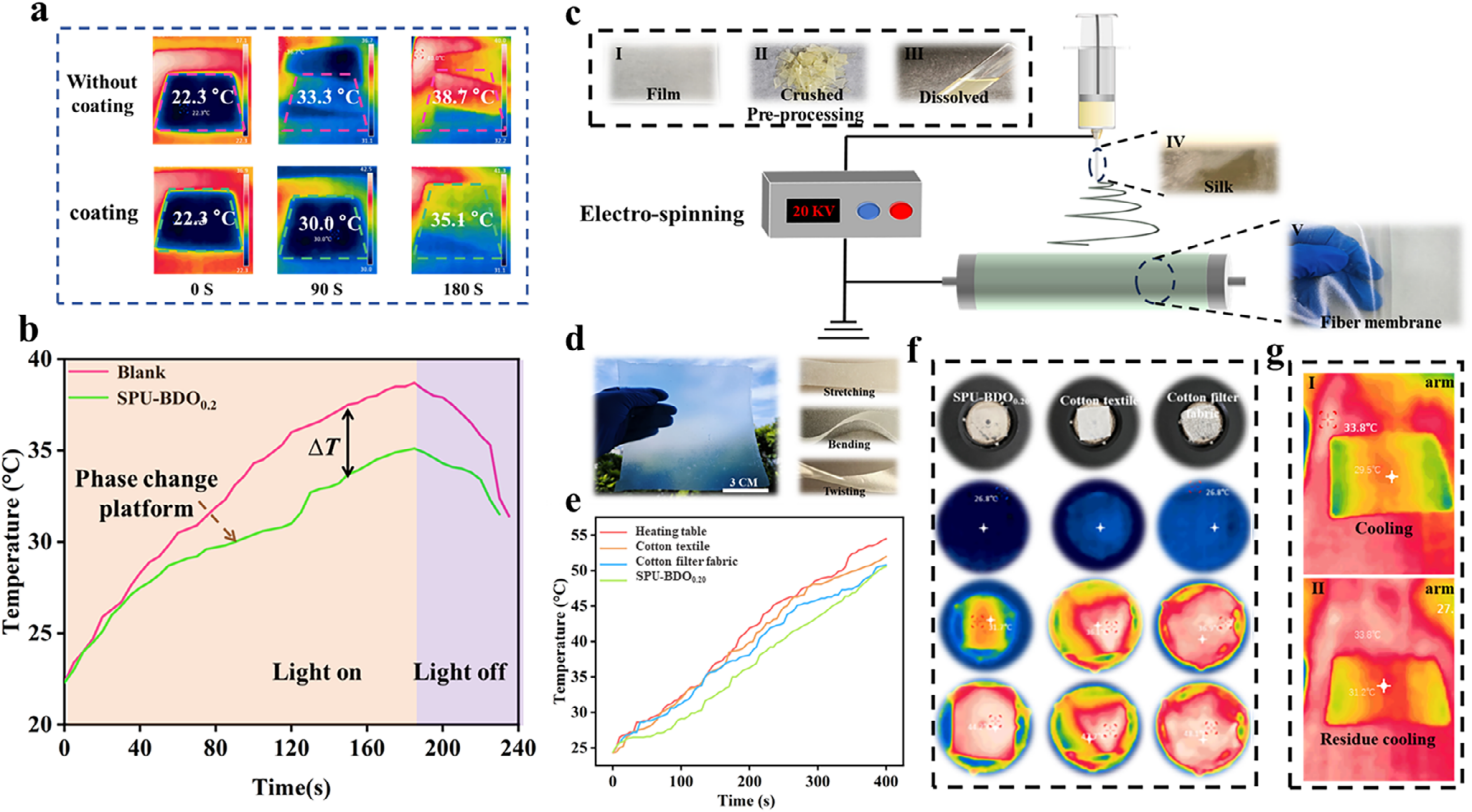
(a) Coating thermal‐buffering experiment with and without SPU‐BDO_0.20_ coating and (b) the corresponding temperature‐time curves. (c) Electrospinning process using dissolved SPU‐BDO_0.20_ solution. (d) Appearance, stretching, bending, and twisting of the electrospinning membranes. (e) Temperature‐time profiles of SPU‐BDO_0.20_, cotton textile, and cotton filter fabric on a heated plate and (f) the corresponding Infrared thermography. (g) Heat storage and release on the arm of SPU‐BDO_0.20_ membrane.

To explore applications beyond direct heat storage, the thermal regulation capabilities of SPU‐BDO_0.20_ were evaluated in two key scenarios: as a passive thermal‐buffering coating and as a wearable textile [29]. The thermal‐buffering performance of SPU‐BDO_0.20_ was first investigated under controlled illumination. As shown in Figure 7a, a bare glass substrate (control group) and a glass substrate coated with a 0.5 mm SPU‐BDO_0.20_ film (experimental group) were irradiated for 180 s. The temperature of the control group rises rapidly to 38.7°C, while the SPU‐BDO_0.20_‐coated substrate exhibits a significantly attenuated temperature rise, stabilizing at 35.1°C. The corresponding temperature‐time profiles reveal distinct behaviors: the control shows a continuous temperature increase, whereas the coated sample displays a characteristic temperature plateau near 30°C due to the absorption of latent heat during the solid‐solid phase transition (Figure 7b). Upon removing the light source, the coated sample also cooled more gradually, further confirming the release of stored latent heat. These findings demonstrate the effectiveness of SPU‐BDO_0.20_ for passive thermal buffering in thermal management applications [30].

Given its suitable phase change temperature and inherent flexibility, SPU‐BDO_0.20_ is highly promising for wearable thermal management applications. Electrospinning fibrous membranes were successfully fabricated from SPU‐BDO_0.20_ (Figure 7c) and evaluated as thermoregulatory textiles. The resulting membranes are semi‐translucent, flexible, and mechanically robust, maintaining structural integrity under stretching, bending, and twisting (Figure 7d). The thermal regulation performance of SPU‐BDO_0.20_ membrane was compared to common cotton textile and cotton filter fabric on a heated stage at 55°C. Infrared thermography (Figure 7e,f) shows that the surface temperatures of cotton and gauze increase rapidly, as indicated by their quick color shift from blue to red. In contrast, the SPU‐BDO_0.20_ membrane exhibits a delayed and moderated thermal response, with a slower color transition and a surface temperature consistently 3°C–4°C lower than the conventional cotton controls under identical conditions [31]. Further in‐situ evaluation under simulated harsh environments (35°C ambient) demonstrated practical efficacy. When placed on the arm, SPU‐BDO_0.20_ membrane maintains a stable surface temperature of 29.5°C, despite adjacent skin temperatures rising to 33.8°C (Figure 7g). Persistent cooling effect can be observed after removing the membrane, evidenced by the residual temperature differential. These results confirm the outstanding capability of the SPU‐BDO_0.20_ phase change membrane to regulate microclimate temperature, enabling sustained thermal comfort and highlighting its great potential for advanced thermal management systems.

## 3 Conclusion

In summary, a series of intrinsically flexible solid‐solid polyurethane‐based PCMs (PU‐PCMs) were successfully constructed by introducing crystallizable C18 alkyl side chains into the polymer network, effectively decoupling mechanical performance from energy storage capacity. The hard segments formed by BDO and HDI ensure a robust physical network, while the hanging C18 side chains enable solid–solid phase transition and latent heat storage. By adjusting the BDO‐to‐SDEA molar ratio, the optimized material, SPU‐BDO_0.20_, displays a desirable combination of high toughness (26.9 MJ m^−3^), excellent flexibility (elongation at break of 647.5%), and stable phase change enthalpy (51.1 J g^−1^) within the human comfort temperature range. SPU‐BDO_0.20_ also demonstrates outstanding thermal stability up to 200°C and retains its phase‐change performance and mechanical flexibility after 500 heating‐cooling cycles. Furthermore, electrospun fibrous membranes fabricated from SPU‐BDO_0.20_ show effective thermal regulation in wearable thermal management applications, maintaining a surface temperature 3°C–4°C lower than conventional cotton controls. This molecular design strategy offers a new inspiration for developing high‐performance, flexible, and leakage‐free PCMs for advanced thermal management systems.

## 4 Experimental Section

### 4.1 Materials

2,2'‐(octadecylimino)diethanol (SDEA, 98%) and hexamethylene diisocyanate (HDI, 99%) were purchased from Macklin (Shanghai, China). 1,4‐butanediol (BDO, 99%) was purchased from Meryer (Shanghai, China). Dibutyltin dilaurate (DBTDL, 98%) was purchased from Tianjin Fuchen Reagent (Tianjin, China). Methyl ethyl ketone (MEK, ≥ 99.5%) was purchased from Tianjin Damao Chemical Reagents (Tianjin, China). BDO was further purified in a vacuum oven at 80°C for 12 h before use. All other chemicals used were analytical grade reagents and used without further purification.

### 4.2 Preparation of Polyurethane (SPU‐BDO_x_)

All SPU‐BDO_x_ samples were synthesized with varying molar ratios of BDO to SDEA. A typical synthesis procedure for SPU‐BDO_0.2_ was as follows. First, SDEA and HDI were weighed and added into a 20 mL sample bottle. Then, appropriate amount of MEK was added to dissolve the mixture, followed by the addition of 20 µL DBTDL as a catalyst. The reaction was allowed to proceed at 75°C for 2 h. After that, BDO was added for chain extension, and the reaction continued for another 2 h. Finally, the polyurethane solution was poured into a silica glass mold, and was dried under vacuum at 50°C for 3 h in order to remove the residual solvent and air bubbles. The polyurethane film was then vacuum dried at 80°C for 18 h and at 100°C for 3 h. Other samples were prepared following the same procedure and designated as SPU‐BDO_x_, where x was the molar ratio of the hydroxyl amount of BDO to the total hydroxyl amount of SDEA and BDO. The molar ratio of the total hydroxyl to isocyanate molar amount was 1:1.05 to ensure complete hydroxyl consumption. For example, SPUs with hydroxyl molar ratios ranging from 0.15:0.85, 0.20:0.80, 0.25:0.75, 0.30:0.70, and 0.35:0.65 were named as SPU‐BDO_0.15_, SPU‐BDO_0.20_, SPU‐BDO_0.25_, SPU‐BDO_0.30_, and SPU‐BDO_0.35_, respectively.

### 4.3 Preparation of Fibrous Membrane

The fibrous membrane was prepared by electrospinning. The precursor solution was obtained by dissolving SPU‐BDO_0.20_ in DMF solvent and stirred for 2 h to obtain a homogeneous yellow spinning solution. The obtained solution was loaded into a 20 mL syringe fitted with an 18‐gauge needle. The distance from the tip of the needle to the collector was maintained at 15 cm, with an injection rate of 3.0 mL h^−1^, a steady voltage of 20 kV, and an ambient temperature of 25 ± 5°C. Finally, a white fibrous membrane was obtained.

### 4.4 Characterizations

The chemical structure of the polyurethane films was characterized using attenuated total reflection Fourier transform infrared (ATR‐FTIR, Thermo Fisher, USA) with a scanning range of 4000–500 cm^−1^. Variable‐temperature FTIR spectra were recorded in the temperature ranging from 30°C to 120°C with a heating rate of 10°C min^−1^.

The ^1^H nuclear magnetic resonance (^1^H NMR) spectra were recorded by a Bruker AV 600 m spectrometer (Germany) using deuterated chloroform as solvent.

The gel permeation chromatography (GPC, 2414 RI detector) was used to measure the molecular weight and polydispersity index of the samples. The mobile phase in the GPC system was chromatographically pure tetrahydrofuran. The mobile phase flow rate was maintained at 0.3 mL min^−1^ and the column temperature was kept at 30°C during the test.

The transparency of samples (6 cm diameter, 0.5 mm thickness) was measured on a DU 800 UV–vis spectrophotometer (Beckman Coulter, USA) with wavelength of 400–800 nm using air as reference. The same sample was tested three times in parallel, and the most representative curve was selected.

The hydrophilicity was evaluated by a contact angle analyzer (FTA‐1000 B, First Ten Angstrom Company, USA). A 2 µL water droplet was deposited on the surface of the film, and the angle between the water droplet and the surface of the film was observed. Each sample was tested at least three times and the results were averaged.

X‐ray Diffraction (XRD) patterns were recorded using a x‐ray diffractometer instrument (XD‐2X/M4600, China). The data were collected within a 2*θ* range of 10°–60° with a scanning rate of 10° min^−1^.

Thermal gravimetric analysis (TGA, NETZSCH‐STA 449C) was performed to evaluate the thermal properties. 5–10 mg sample was heated from 30°C to 700°C at a heating rate of 10°C min^−1^ under a N_2_ atmosphere.

Low‐field NMR (LF‐NMR) was performed on a VTMR20‐010V‐I model spectrometer (Newman, China). The samples were cut into 15 mm × 5 mm strips and placed in quartz tubes. The *T*_2_ time profiles of the test samples were performed at room temperature using a CMPMG‐VD sequence with a cumulative number of 10 and a sampling bandwidth of 500 KHz.

### 4.5 Mechanical Test

The mechanical properties of SPU‐BDO_x_ (with a size of 30 mm × 10 mm) were measured by an electronic universal testing machine (UTM 5504). The testing was carried out at a tensile rate of 50 mm min^−1^. Each sample was tested at least three times to obtain the corresponding mechanical property data.

The trouser tear test was carried out in the Sansi universal electronic testing machine (UTM5504) at room temperature. The testing samples were cut into a rectangular shape (length of 50 mm, width of 15 mm, and thickness of 0.5 mm), with a tear of 20 mm in the length of the film at the tear point. The tearing test was performed at a tensile rate of 50 mm min^−1^, while the tearing force *F* was recorded. The tear energy was calculated by the following Equation (2) [32]:

(2) Tearenergy=2F/t

where *F* (N) is the constant tensile force and *t* (mm) is the thickness.

The fracture energy test was conducted by the tensile test using the single‐edge notched sample with a slit length of 1 mm. Both notched and unnotched samples (length of 10 mm, width of 5 mm, and thickness of 0.5 mm) were tested at the stretching speed of 10 mm min^−1^, corresponding to a strain rate of 20 mm min^−1^. The fracture energy (*G*_c_) was calculated by the following Equation (3):

(3) $$G_{c}=\frac{6\mathrm{Wc}}{\sqrt{\lambda_{c}}}\;$$

where 𝑐 is the length of the slit (1 mm), *λ*_c_ is the elongation at break of the notched sample, *W* is the strain energy calculated by integration of the stress‐strain curve of the unnotched specimen until 𝜀_𝑐_ (𝜀_𝑐_ = *λ*_c_‐1).

### 4.6 Phase Transition Measurement

For the leakage test, SPU‐BDOx films were cut into square specimens with a size of approximately 10 mm × 10 mm and a thickness of approximately 0.5 mm. Each specimen was placed on clean qualitative filter paper and heated on a temperature‐controlled hot stage. The samples were first photographed at 30°C and then heated to 80°C for 30 min, followed by optical photographs to evaluate shape retention and leakage behavior. Leakage was identified by visible wetting, oil‐like stains, or penetration marks on the filter paper. Pure SDEA was used as a control sample.

A dynamic thermomechanical analyser (Netzsch DMA 242E) was used to determine the energy storage modulus and loss modulus of SPU‐BDO_x_. The samples were cut into 20 mm × 5 mm strips for testing. The temperature was reduced to −70°C using liquid nitrogen and then heated to 120°C at 5°C min^−1^ with a tensile frequency of 1 Hz.

The phase transition temperature and enthalpy of SPU‐BDO_x_ were analyzed using a differential scanning calorimeter (DSC, PerkinElmer DSC 6000). During the test, a nitrogen atmosphere was employed. The sample was first heated from 30°C to 120°C at a heating rate of 10°C min^−^^1^ to eliminate thermal history. Subsequently, it was cooled to −70°C, and then reheated to 120°C at the same heating rate.

An infrared thermography camera (UNI‐T, UTi120S) was used to monitor the temperature changes of cotton, gauze, and SPU‐BDO_0.20_ fibrous membranes during the heating process. Thermal images were captured every 5 s, and temperature‐time curves were generated using UNI‐T (UTi120S) software.

## Author Contributions

**Huizhou Luo**: methodology, software, data curation, investigation, validation, formal analysis, writing – original draft. **Henghui Deng**: investigation, methodology. **Jun Zhang**: investigation, validation, formal analysis. **Yongyin Zhu**: investigation, validation. **Chaoqun Zhang**: conceptualization, methodology, funding acquisition, resources, project administration, writing – review and editing. **Zehong Chen**: conceptualization, methodology, writing – original draft, writing – review and editing, supervision. **Hebo Shi**: investigation, validation, data curation, formal analysis, writing – original draft, visualization.

## Funding

This work was sponsored by the Guangdong Forestry Science and Technology Innovation Project (2024KJCX003), National Key Research and Development Program of China (2023YFD1800105), National Natural Science Foundation of China (32222057), Guangdong Basic and Applied Basic Research Foundation (2024B1515040004 and 2026A1515011725), Guangzhou Science and Technology Plan Project (2024A04J6354).

## Conflicts of Interest

The authors declare no conflicts of interest.

## Supporting information




**Supporting File**: advs76170‐sup‐0001‐SuppMat.docx.

## Data Availability

The data that support the findings of this study are available from the corresponding author upon reasonable request.
