# Supplementary material for: Breaking the Trade‐Off of Mechanical Robustness and Energy Storage Capacity in Phase Change Materials Through a Molecular Design Strategy of Hard‐Segment‐Anchored/Side‐Chain‐Storage
Source: Adv Sci (Weinh). 2026 Jun 15;13(51):e76170. doi: 10.1002/advs.76170 (PMC13336753; doi:10.1002/advs.76170)
Supplement: Supplementary file 1 [file ADVS-13-e76170-s001.docx]

Supporting Information

Breaking the trade-off of mechanical robustness and energy storage capacity in phase change materials through a molecular design strategy of hard-segment-anchored/side-chain-storage

Huizhou Luo , Hebo Shi, Jun Zhang , Henghui Deng, Yongyin Zhu, Zehong Chen*, and Chaoqun Zhang *

H. Luo , H. Shi, J. Zhang , H. Deng, Y. Zhu, Z. Chen, C. Zhang

College of Future Biomass, South China Agricultural University, 483 Wushan Road, Guangzhou 510642, China.
Corresponding author. E-mail: chenzehong24@scau.edu.cn (Z. Chen)

C. Zhang
College of Material Science and Art Design, Inner Mongolia Agricultural University, Hohhot 010018, China.

Corresponding author. E-mail: zhangcq@scau.edu.cn, nwzcq@gmail.com (C. Zhang)

H. Luo and H. Shi contributed equally to this work.

**Figure S1.** ^1^H NMR spectrum of SPU-BDO_0.20_ (400 MHz, CDCl₃-*d*6).

**Figure S2.** GPC curves of SPU-BDO_x_. Eluent: THF.

**Figure S3.** Comparison of SPU-BDO_0.20_ with other reported flexible PCMs in terms of phase-change enthalpy and elongation at break.

**Figure S4.** The damping efficiencies of SPU-BDO_0.20_ at strains of 50%, 100%, 150%, 200%, 250%, and 300%.

**Table S1.** Summary of *M*_n_, *M*_w_, and dispersity (*M*_w_/*M*_n_) of SPU-BDO_x_.

| **Sample** | ***M*_n_** | ***M*_w_** | ***M*_w_/*M*_n_** |
| --- | --- | --- | --- |
| SPU-BDO_0.15_ | 14128 | 22421 | 1.59 |
| SPU-BDO_0.20_ | 14996 | 22512 | 1.50 |
| SPU-BDO_0.25_ | 13462 | 21653 | 1.61 |
| SPU-BDO_0.30_ | 13875 | 22034 | 1.59 |
| SPU-BDO_0.35_ | 14427 | 22817 | 1.58 |

**Table S2.** Summary of mechanical properties of SPU-BDO_x_.

| **Sample** | **Strength**  **(MPa)** | **Elongation at break (%)** | **Toughness**  **(MJ m^-3^)** | **Young’s modulus (MPa)** |
| --- | --- | --- | --- | --- |
| SPU-BDO_0.15_ | 2.5±0.06 | 567.5±29.77 | 12.3±2.41 | 26.8±5.19 |
| SPU-BDO_0.20_ | 5.9±0.38 | 647.5±65.76 | 26.9±6.36 | 82.9±3.04 |
| SPU-BDO_0.25_ | 3.1±0.12 | 426.0±41.79 | 9.9±2.88 | 37.3±11.24 |
| SPU-BDO_0.30_ | 6.3±0.85 | 304.0±25.06 | 13.9±0.21 | 134.1±8.34 |
| SPU-BDO_0.35_ | 10.3±0.99 | 50.8±9.21 | 7.5±0.28 | 160.4±14.28 |

**Table S3.** Summary of phase transition data about SDEA and SPU-BDO_x_.

| **Sample** | **Heating process** | | **Cooling process** | |
| --- | --- | --- | --- | --- |
|  | ***T*_m_ (℃)** | ***∆H*_m_ (J g^-1^)** | ***T*_c_ (℃)** | ***∆H*_c_ (J g^-1^)** |
| SDEA | 46.7 | 90.4 | 38.4 | 95.1 |
| SPU-BDO_0.15_ | 30.9 | 50.7 | 16.7 | 52.4 |
| SPU-BDO_0.20_ | 29.2 | 50.4 | 14.1 | 51.8 |
| SPU-BDO_0.25_ | 27.3 | 49.4 | 13.1 | 51.5 |
| SPU-BDO_0.30_ | 25.5 | 47.5 | 10.0 | 50.3 |
| SPU-BDO_0.35_ | 24.9 | 45.4 | 9.8 | 49.6 |

**Table S4.** Phase-change and mechanical properties of SPU-BDO_0.20_ before and after 500 cycles.

| **Properties** | | **Uncycled** | **After 500 cycles** |
| --- | --- | --- | --- |
| Heating process | *T*_m_ (℃) | 29.2 | 29.0 |
|  | *∆H*_m_ (J g^-1^) | 50.4 | 48.8 |
| Cooling process | *T*_c_ (℃) | 14.1 | 13.7 |
|  | *∆H*_c_ (J g^-1^) | 51.8 | 50.6 |
| Tensile strength (MPa) | | 5.5 | 4.8 |
| Elongation at break (%) | | 698.3 | 679.6 |
| Toughness (MJ m^-3^) | | 26.9 | 24.2 |
| Young’s modulus (MPa) | | 82.9 | 78.6 |

**Table S5.** Comparison of SPU-BDO_0.20_ with other reported flexible PCMs in terms of phase-change enthalpy and elongation at break.

| **PCMs** | **Δ*H***  **(J g^-1^)** | **Elongation at break (%)** | **Ref.** |
| --- | --- | --- | --- |
| MPCM/SEPS | 60.6 | 310 | Compos. Part A, 2022, 163: 107203 |
| PU-SSPCM | 61.48 | 420 | J. Energy Storage, 2022, 52: 104751 |
| Zn-SEPDM/CP@DA | 29.8 | 617 | Polym. Eng. Sci., 2025, 65: 5523 |
| PBHA-C5/PBT | 43.9 | 50 | Polymers, 2022, 14(16): 3298 |
| PEG/PCDL/PTMG-PU | 54.01 | 45.33 | Res. Sq., 2023, rs-3607955 |
| OP-Ag-nAgMWNT | 7.44 | 0.8 | Adv. Mater., 2023, 35: 2300956 |
| TPN/TPN-BNx | 43.6 | 5.7 | Chem. Eng. J., 2025, 510: 161680 |
| 30 wt% PEG/CA | 25.96 | 8 | Appl. Energy 2011, 88: 3133 |
| DGEM-18/FA/MA/0.1BW | 52.7 | 25 | Chem. Eng. J., 2022, 448: 137749 |
| SPU-BDO_0.20_ | 51.1 | 647.5 | This work |

**References**

1. Chen, Changzhong, Linge Wang, and Yong Huang. “Electrospun phase change fibers based on polyethylene glycol/cellulose acetate blends.” Applied Energy 88.9 (2011): 3133-3139, https://doi.org/10.1016/j.apenergy.2011.02.026.
2. Abdul Jaleel, Shabas Ahammed, Taehun Kim, and Seunghyun Baik. “Covalently functionalized leakage‐free healable phase‐change interface materials with extraordinary high‐thermal conductivity and low‐thermal resistance.” Advanced Materials 35.30 (2023): 2300956, https://doi.org/10.1002/adma.202300956.
3. Koo, Jahyeon, et al. “Thermally Conductive and Self-Healable Phase Change Nanocomposites for Advanced Thermal Management Systems.” Chemical Engineering Journal 510 (2025): 161680, https://doi.org/10.1016/j.cej.2025.161680.
4. Liu, Ziyu, et al. “Bio-based recyclable Form-Stable phase change material based on thermally reversible Diels–Alder reaction for sustainable thermal energy storage.” Chemical Engineering Journal 448 (2022): 137749, http://dx.doi.org/10.1016/j.cej.2022.137749.
5. Ma, Yanqi, et al. “Flexible phase change composite films with improved thermal conductivity and superb thermal reliability for electronic chip thermal management.” Composites Part A: Applied Science and Manufacturing 163 (2022): 107203, https://doi.org/10.1016/j.compositesa.2022.107203.
6. Liao, Yanning, et al. “Super-elastic and shape-stable solid-solid phase change materials for thermal management of electronics.” Journal of Energy Storage 52 (2022): 104751, https://doi.org/10.1016/j.est.2022.104751.
7. Liu, Mingshuo, et al. “Long‐Term Form‐Stable Phase Change Materials Based on Ionomers and Fatty Acid Eutectics for Thermal Management.” Polymer Engineering & Science 65.10 (2025): 5523-5534, https://doi.org/10.1002/pen.70085.
8. Lan, Tsung-Yu, et al. “A rapid thermal absorption rate and high latent heat enthalpy phase change fiber derived from bio-based low melting point copolyesters.” Polymers 14.16 (2022): 3298, https://doi.org/10.3390/polym14163298.
9. Cui, Shanshan, et al. “Phase change and thermal energy storage properties of polyurethane phase change materials with mixed soft segment and crosslinking structure.” Research Square (2023), rs-3607955, https://doi.org/10.21203/rs.3.rs-3607955/v1.
